# Melting of generalized Wigner crystals in transition metal dichalcogenide heterobilayer Moiré systems

**DOI:** 10.1038/s41467-022-34683-x

**Published:** 2022-11-19

**Authors:** Michael Matty, Eun-Ah Kim

**Affiliations:** Department of Physics, Cornell University, Ithaca, NY 14853 USA

**Keywords:** Electronic properties and materials, Two-dimensional materials

## Abstract

Moiré superlattice systems such as transition metal dichalcogenide heterobilayers have garnered significant recent interest due to their promising utility as tunable solid state simulators. Recent experiments on a WSe_2_/WS_2_ heterobilayer detected incompressible charge ordered states that one can view as generalized Wigner crystals. The tunability of the transition metal dichalcogenide heterobilayer Moiré system presents an opportunity to study the rich set of possible phases upon melting these charge-ordered states. Here we use Monte Carlo simulations to study these intermediate phases in between incompressible charge-ordered states in the strong coupling limit. We find two distinct stripe solid states to be each preceded by distinct types of nematic states. In particular, we discover microscopic mechanisms that stabilize each of the nematic states, whose order parameter transforms as the two-dimensional *E* representation of the Moiré lattice point group. Our results provide a testable experimental prediction of where both types of nematic occur, and elucidate the microscopic mechanism driving their formation.

## Abstract (web summary)

Recent experiments on a WSe_2_/WS_2_ hetero-bilayer detected incompressible charge ordered states considered to be generalized Wigner crystals. Here, by performing Monte Carlo simulations of a triangular moiré lattice, the authors study the phases which emerge on melting such charge-ordered states in partially filled moiré bands, finding two distinct nematic states and a hexagonal domain wall state.

## Introduction

**Fig. 1:**
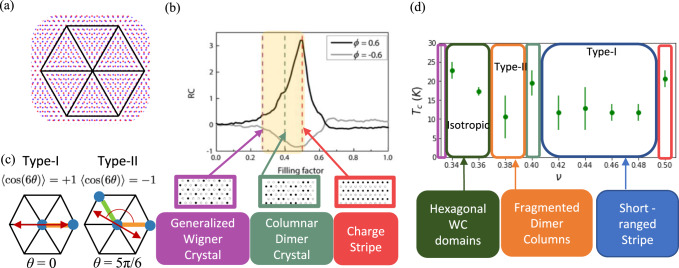
Electronic states in TMD Moiré systems. **a** The red and blue dots show the sites of two honeycomb lattices whose lattice constants differ by 5%. These lattices are layered at zero twist-angle, resulting in an emergent triangular Moiré lattice with a unit cell indicated by the black lines. In the case of TMD heterobilayers, the Moiré lattice has point group *C*_3_. **b** Top: optical anisotropy as a function of Moiré lattice filling, reproduced with permission from ref. 16. Bottom: charge order patterns at 1/3-, 2/5-, and 1/2-filling as determined by Monte Carlo, reproduced with permission from ref. 7. **c** Here we show the Moiré unit cell with occupied lattice sites represented by blue dots, and the bond connecting nearest neighbor pairs colored according to its orientation. On a lattice with *C*_3_ symmetry, there are two distinct types of nematic states. Type-I nematics (left) have a nematic director oriented along a single majority bond orientation at *θ* ∈ {0, *π*/3, 2*π*/3} and have $\langle \cos (6\theta )\rangle=1$. Type-II nematics (right) have a nematic director oriented perpendicular to a single minority bond orientation at *θ* ∈ {*π*/6, *π*/2, 5*π*/6} and have $\langle \cos (6\theta )\rangle=-1$. **d** The critical temperature as a function of Moiré lattice filling as determined by Monte Carlo. At 1/3-, 2/5-, and 1/2-filling we find the same charge-ordered states as in (**b**). Between 2/5-filling and 1/2-filling we find a type-I nematic state defined by short-range domains of the 1/2-filled charge stripe state. Above 1/3-filling, we find an isotropic state defined by hexagonal domains of the 1/3 generalized Wigner crystal, which eventually gives way to a type-II nematic state defined by fragmented domains of the 2/5-filled columnar dimer crystal. For the two isotropic phases we determine *T*_*c*_ from the integrated peak weight of the structure factor. For the remaining anisotropic phases we use the jump in the nematic correlation function.

The promise of a highly tunable lattice system that can allow solid-state-based simulation of strong coupling physics^1–3^ has largely driven the explosion of efforts studying Moiré superlattices. The transition metal dichalcogenide (TMD) heterobilayer Moiré systems with zero twist-angle (see Fig. 1a) and localized Wannier orbitals form a uniquely simple platform to explore phases driven by strong interactions^4–6^. Many theoretical efforts went into the study of charge order in TMD moiré systems at commensurate fillings. Specifically, incompressible charge-ordered states at commensurate fillings have been theorized based on Monte Carlo simulations^6–8^ and Hartree-Fock calculations^9^. Moreover, refs. 10, 11 discuss predictions of specific experimental systems capable of realizing such states based on estimated material parameters. These incompressible charge-ordered states have been detected experimentally in a WS_2_/WSe_2_ system as well^5–7,12^. However, an understanding of the phase diagram when one tunes the density away from the incompressible states into the compressible region is still lacking.

The incompressible charge orders can be viewed as generalized Wigner crystalline states that reduce the symmetry of the underlying moiré lattice, as they are driven by the long-range Coulomb interaction. The density-controlled melting of Wigner crystals is expected to result in a rich hierarchy of intermediate phases^13–15^. While a microscopic theoretical study of Wigner crystal melting is challenging due to the continuous spatial symmetry, the melting of generalized Wigner crystals is more amenable to a microscopic study due to the lattice. The observation of intermediate compressible states with optical anisotropy^16^ (see Fig. 1b) and the tunability beyond density^17,18^ present a tantalizing possibility to study melting and the possible intermediate phases of the generalized Wigner crystals.

The underlying lattice in the generalized Wigner crystal reduces the continuous rotational symmetry to *C*_3_ point group symmetry. Reference 19 studied the melting of a 1/3-filled crystalline state on a triangular lattice in the context of Krypton adsorbed on Graphene. Based on the free energy costs of the domain walls and domain wall intersections, they reasoned that the generalized WC would first melt into a hexagonal liquid, and then crystallize into a stripe solid. From the modern perspectives of electronic liquid crystals^20^, one anticipates nematic fluid states in the vicinity of crystalline anisotropic states such as the stripe solid. Moreover, the *C*_3_ point group symmetry of the triangular lattice relevant for hetero-TMD Moiré sytems further enriches the possibilities of the intermediate fluid phases. The triangular lattice admits two types of nematic states due to the nematic order parameter transforming as a 2-dimensional irreducible representation of the lattice point group^21,22^. The hetero-TMD Moiré systems present an excellent opportunity to study these intermediate liquid phases.

As the quantum melting of charge order is a notoriously difficult problem^23^, in this paper we will take advantage of the small bandwidth in hetero-TMD systems and use a strong coupling approach. We study Monte Carlo simulations inspired by hetero-TMD Moiré systems at and between commensurate charge-ordered states. We analyze our results in terms of the structure factor and a nematic order parameter correlation function. In particular, we distinguish between the two possible types of nematic states illustrated in Fig. 1c, one associated with the director aligned with a single majority bond orientation (which we dub type-I), and the other perpendicular to a single minority bond orientation (type-II). As shown in Fig. 1d, we find the type-I nematic and type-II nematic to each robustly appear between 2/5 and 1/2 and 1/3 and 2/5, respectively. We conclude with a discussion.

## Results

As the orientation of the nematic director is defined within the angle range *θ* ∈ [0, *π*) (Fig. 1c) we define the local nematic field using complex notation *N*(**r**) = ∣*N*(**r**)∣*e*^*i*2*θ*(**r**)^. In terms of this nematic order parameter field, the free energy density describing the isotropic-nematic transition in a trigonal system takes the following form^22,24–26^:

1 $$f[N({{{{{{{\bf{r}}}}}}}})]=\frac{r}{2}|N({{{{{{{\bf{r}}}}}}}}){|}^{2}+\frac{u}{4}|N({{{{{{{\bf{r}}}}}}}}){|}^{4}+\frac{\gamma }{3}|N({{{{{{{\bf{r}}}}}}}}){|}^{3}\cos (6\theta ({{{{{{{\bf{r}}}}}}}})).$$

As usual, *r* changing sign signifies a transition into nematic order with positive definite *u* for stability. The cubic term is allowed by symmetry in a trigonal system, and is not allowed in a tetragonal system. Clearly, the sign of *γ* will determine the expectation value of $\cos (6\theta )$, and thus two types of nematic: type-I (*γ* < 0 and thus $\langle \cos (6\theta )\rangle=+ \!1$) and type-II ($\gamma \, > \,0,\langle \cos (6\theta )\rangle=-\!1$).

We explore the phase diagram using classical Monte Carlo as a function of temperature *T* and the number of particles per Moiré site *ν*. To emulate the experimental setup in refs. 5, 7, 16, 27, the Hamiltonian that we simulate describes the Coulomb interaction for electrons halfway between two dielectric gates a distance *d* apart with dielectric constant *ϵ*:

2 $$\begin{array}{lll}{{{{{{{\mathcal{H}}}}}}}}=\frac{1}{2}\mathop{\sum}\limits_{i\ne j}\rho ({{{{{{{{\bf{r}}}}}}}}}_{i})\rho ({{{{{{{{\bf{r}}}}}}}}}_{j})\left(\frac{{e}^{2}}{4\pi \epsilon {\epsilon }_{0}a}\right)\frac{4}{d}\\ \quad\quad\times \left[\mathop{\sum }\limits_{n=0}^{\infty }{K}_{0}\left(\frac{\pi (2n+1)|{{{{{{{{\bf{r}}}}}}}}}_{{{{{{{{\bf{i}}}}}}}}}-{{{{{{{{\bf{r}}}}}}}}}_{{{{{{{{\bf{j}}}}}}}}}|}{d}\right)\right].\end{array}$$

Here, *K*_0_ is the modified Bessel function of the second kind, *a* is the Moiré lattice constant, and *ρ*(**r**_*i*,*j*_) ∈ {0, 1} are the occupancies of lattice sites *i*, *j*. As in refs. 7, 16, we take *a* = 8*nm* and *d* = 10*a*, and we take *e*^2^/(4*πϵϵ*_0_*a*) as our unit of energy for simulation. For further simulation details see the “Methods” section and SI section 1.

At each point in phase space, we calculate the Monte Carlo average of the structure factor

3 $$\langle S({{{{{{{\bf{Q}}}}}}}})\rangle=\frac{1}{{\ell }^{4}}\left\langle \mathop{\sum}\limits_{i,j}\rho ({{{{{{{{\bf{r}}}}}}}}}_{i})\rho ({{{{{{{{\bf{r}}}}}}}}}_{j}){e}^{-i{{{{{{{\bf{Q}}}}}}}}\cdot ({{{{{{{{\bf{r}}}}}}}}}_{i}-{{{{{{{{\bf{r}}}}}}}}}_{j})}\right\rangle,$$

to assess crystalline order. To assess the degree of rotational symmetry breaking, we also calculate the average of the nematic order parameter correlation function given by

4 $$\frac{1}{{\ell }^{4}}\mathop{\sum}\limits_{{{{{{{{\bf{r}}}}}}}},{{{{{{{\bf{r}}}}}}}}^{\prime} }\langle C({{{{{{{\bf{r}}}}}}}},{{{{{{{\bf{r}}}}}}}}^{\prime} )\rangle=\frac{1}{{\ell }^{4}}\mathop{\sum}\limits_{{{{{{{{\bf{r}}}}}}}},{{{{{{{\bf{r}}}}}}}}^{\prime} }\langle N({{{{{{{\bf{r}}}}}}}}){N}^{*}({{{{{{{\bf{r}}}}}}}}^{\prime} )\rangle=\frac{1}{{\ell }^{4}}\langle \tilde{C}({{{{{{{\bf{q}}}}}}}}=0)\rangle$$

where **q** denotes Fourier momentum. At high temperatures, when 〈*N*(**r**)〉 = 0, $\langle \tilde{C}({{{{{{{\bf{q}}}}}}}}=0)\rangle /{\ell }^{4}$ behaves as *k*_*B*_*T* times the nematic susceptibility: *χ*(**q** = 0)*k*_*B*_*T*. Generically we expect this to have some continuous behavior as a function of temperature. However, when the order parameter develops an expectation value in a nematic state, $\langle \tilde{C}({{{{{{{\bf{q}}}}}}}}=0)\rangle /{\ell }^{4}$ should acquire a constant, non-zero value. To determine the type of nematicity exhibited by nematic states, we also calculate $\langle \cos (6\theta )\rangle$, where, as in Fig. 1c, type-I (type-II) nematic states have $\langle \cos (6\theta )\rangle=+ 1\,(-1)$. For further details about the calculation of these quantities from our Monte Carlo simulation data including a formulation of the nematic order parameter in terms of the density operators *ρ*(**r**), see SI section 2. All results that we show are obtained from an *ℓ* = 20 system, except for exactly at *ν* = 1/3 since 20 × 20/3 is not an integer. In all cases, we perform 10^5^ updates per site for equilibration at each temperature, and then 2 × 10^5^ updates per site for data collection.

**Fig. 2:**
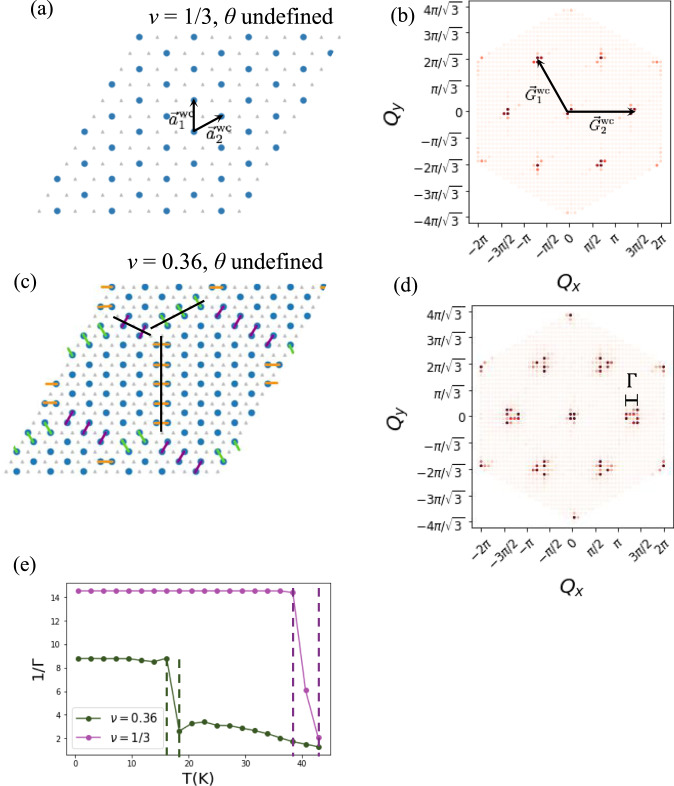
Isotropic states. **a** Generalized Wigner crystal at *ν* = 1/3 particles per Moiré site for an *ℓ* = 12 system obtained by Monte Carlo. The system is isotropic and thus the orientation of the nematic director, *θ*, is undefined. **b** The Monte Carlo average of the structure factor at *ν* = 1/3. The structure factor exhibits peaks at the reciprocal lattice vectors of the *ν* = 1/3 generalized Wigner crystal. **c** Monte Carlo equilibrated state at *ν* = 0.36 showing the isotropic hexagonal Wigner crystal domain state. Nearest neighbor bonds are shown and color-coded according to their orientation. Domain walls (marked with black lines) between the three registries of the generalized Wigner crystal state meet at 2*π*/3 angles, forming hexagonal domains. **d** The Monte Carlo average of the structure factor at *ν* = 0.36, showing the short-range correlated nature of the hexagonal Wigner crystal domain state in the broadened peaks as compared to (**b**). The peak width Γ in (**e**) is calculated along the line *Q*_*y*_ = 0. **e** The Monte Carlo average of the reciprocal width 1/Γ of the peak at ${{{{{{{{\bf{G}}}}}}}}}_{2}^{wc}$ for *ν* = 1/3 and *ν* = 0.36. It plateaus below *T*_*c*_ and illustrates the smaller correlation length at *ν* = 0.36 compared to at *ν* = 1/3. Error bars are smaller than the symbol size. Dashed lines correspond to the boundaries of the “jump” in 1/Γ, which we note correspond to the jump boundaries in the integrated peak intensity as well. We assign *T*_*c*_ to be the temperature midway between the dashed lines.

At *ν* = 1/3, we find the isotropic generalized Wigner crystalline phase, shown in Fig. 2a for *ℓ* = 12. This phase has lattice vectors ${{{{{{{{\bf{a}}}}}}}}}_{1}^{{{{{{{{\rm{wc}}}}}}}}}=(0,\,\,\sqrt{3})$ and ${{{{{{{{\bf{a}}}}}}}}}_{2}^{{{{{{{{\rm{wc}}}}}}}}}=(3/2,\,\,\sqrt{3}/2)$ as indicated by the black arrows in Fig. 2a. The structure factor shows well-defined peaks at the reciprocal lattice vectors ${{{{{{{{\bf{G}}}}}}}}}_{1}^{{{{{{{{\rm{wc}}}}}}}}}=(-2\pi /3,\,\,2\pi /\sqrt{3})$) and ${{{{{{{{\bf{G}}}}}}}}}_{2}^{{{{{{{{\rm{wc}}}}}}}}}=(4\pi /3,0)$ associated with the crystalline state (Fig. 2b). Upon increasing the density, this crystalline state starts to melt, but it maintains an isotropic, compressible state to a certain filling. At small fillings away from the 1/3-state, as shown in Fig. 2c, the extra particles form domain walls between the three different registries of the generalized WC state. Three domain walls are marked with black lines in Fig. 2c. The domain walls meet at 2*π*/3 angles, reminiscent of what was found in ref. 19.

As in ref. 19, this domain wall structure is stable while the density of domain walls is dilute (and hence the domain walls are long) because energetics favor 2*π*/3 angles. Taking into account interactions up to fifth neighbor, the energy contributed to the Hamiltonian by the six particles in the three dimers composing the 2*π*/3 vertex is

5 $${E}_{v}=3{V}_{1}+\frac{21}{2}{V}_{2}+6{V}_{3}+\frac{21}{2}{V}_{4}+\frac{15}{2}{V}_{5}$$

while that of the particles composing three straight domain wall dimers is

6 $${E}_{DW}=3{V}_{1}+12{V}_{2}+6{V}_{3}+6{V}_{4}+9{V}_{5},$$

where *V*_*i*_ denotes the energy of two *i*’th neighbor particles. Using Eq. (2) it is easy to check that *E*_*DW*_ − *E*_*v*_ > 0, and thus (at least to this order of interaction), it is energetically favorable to have 2*π*/3-vertices, even at low temperatures. However, the densest possible hexagonal domain state consists of a close packing of 2*π*/3-vertices, which has density *ν* = 3/8. Thus, this state certainly cannot exist at densities *ν* > 3/8. In Fig. 2d, the structure factor of this compressible state exhibits broadened superstructure peaks centered at the generalized WC periodicities with the width Γ reflecting finite correlation length limited by the hexagonal domain size. Γ is calculated by a Gaussian fit to the peak along the line *Q*_*y*_ = 0.

The temperature evolution of the inverse peak width 1/Γ, which behaves like the correlation length, establishes a clear distinction between the incompressible phase at *ν* = 1/3 and the compressible hexagonal domain wall state at *ν* = 0.36. The transition into the incompressible generalized Wigner crystal at *ν* = 1/3 is evidenced by the development of a momentum resolution limited sharp peak (see Fig. 2e). At this filling, the ordering phenomena belongs to the universality class of the three-state Potts model^7^ with a second order transition. On the other hand, the correlation length of the hexagonal domain wall state at *ν* = 0.36 exhibits a discontinuous jump reflecting the saturation of the correlation length at a finite value. This transition appears to be first order due to the discontinuous jump in 1/Γ and the integrated peak width, which jumps at the same temperature.

**Fig. 3:**
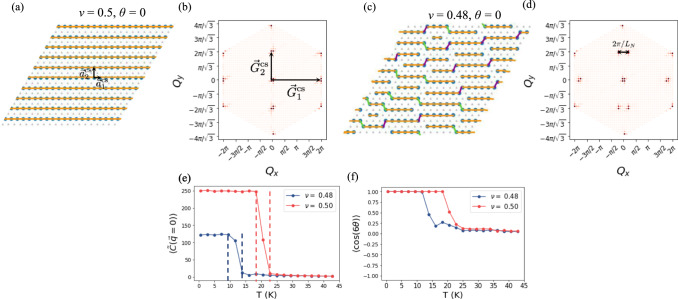
Charge stripe and type-I nematic states. **a** The Monte Carlo charge stripe state at *ν* = 1/2 shown for nematic director orientation *θ* = 0. We annotate nearest neighbor bonds and color them according to their orientation. The red arrows indicate the charge stripe lattice vectors. **b** The Monte Carlo average of the structure factor at *ν* = 1/2, averaged over configurations with director orientation *θ* = 0. The red arrows indicate peaks at the reciprocal lattice vectors of the charge stripe state. **c** Monte Carlo equilibrated state at *ν* = 0.48 showing short-ranged stripe nematic state for nematic director orientation *θ* = 0. We again annotate nearest-neighbor bonds. Pieces of the charge stripe state are separated by dislocations. **d** The Monte Carlo average of the structure factor, at *ν* = 0.48, averaged over configurations with director orientation *θ* = 0. The peak at $(0,2\pi /\sqrt{3})$ splits into two peaks separated by 2*π*/*L*_*N*_ where *L*_*N*_ is the average stripe domain length. **e** The Monte Carlo average of the nematic order parameter correlation function at *ν* = 1/2 and *ν* = 0.48. Error bars are smaller than the symbol size. It jumps to a finite, constant value at *T*_*c*_. Dashed lines denote the boundaries of the jump and we assign *T*_*c*_ to be the temperature midway between the boundaries, while the boundaries give the error bars in Fig. 1d. **f** $\langle \cos (6\theta )\rangle$ at *ν* = 1/2 and *ν* = 0.48, which goes to + 1 at *T*_*c*_ in both cases. This suggests type-I nematicity at both of these fillings. Error bars are smaller than the symbol size.

The state is dramatically different at *ν* = 1/2. We have the charge stripe state shown in Fig. 3a with lattice vectors ${{{{{{{{\bf{a}}}}}}}}}_{1}^{{{{{{{{\rm{cs}}}}}}}}}=(1,0)$ and ${{{{{{{{\bf{a}}}}}}}}}_{2}^{{{{{{{{\rm{cs}}}}}}}}}=(0,\sqrt{3})$. There are two degenerate charge stripe states whose lattice vectors are related by *π*/3 and 2*π*/3 rotations of ${{{{{{{{\bf{a}}}}}}}}}_{1,2}^{{{{{{{{\rm{cs}}}}}}}}}$. The structure factor in Fig. 3b, averaged over configurations with the same orientation as the one shown in Fig. 3a, contains peaks at the reciprocal lattice vectors ${{{{{{{{\bf{G}}}}}}}}}_{1}^{{{{{{{{\rm{cs}}}}}}}}}=(2\pi,\,\,0)$ and ${{{{{{{{\bf{G}}}}}}}}}_{2}^{{{{{{{{\rm{cs}}}}}}}}}=(0,2\pi /\sqrt{3})$. As expected, ${{{{{{{{\bf{G}}}}}}}}}_{i}^{{{{{{{{\rm{cs}}}}}}}}}\cdot {{{{{{{{\bf{a}}}}}}}}}_{j}^{{{{{{{{\rm{cs}}}}}}}}}=2\pi {\delta }_{ij}$. Diluting the 1/2-filled state, the stripes become shorter via the introduction of dislocations, as shown in Fig. 3c. The structure factor reflects the finite length of these stripe domains in the splitting of the stripe peak over the span of the stripe domain size scale. The peak at ${{{{{{{{\bf{G}}}}}}}}}_{2}^{{{{{{{{\rm{cs}}}}}}}}}$ is split into two peaks separated by 2*π*/*L*_*N*_ where *L*_*N*_ ≈ 4.63 is the average stripe domain size. The nematic correlation function reveals that, unlike the isotropic phases in Fig. 2e, $\langle \tilde{C}({{{{{{{\bf{q}}}}}}}}=0)\rangle$ shows a sharp jump at *T*_*c*_ to a finite value in Fig. 3e. This indicates that these are nematic states. By examining $\langle \cos (6\theta )\rangle$ in Fig. 3f, we can see that both of these phases are type-I nematic. The discontinuous jump in the correlation function suggests that these transitions are first-order. In Fig. 1d, we assign the critical temperature to be the center of the jump, and the error bars are given by the width of the jump.

**Fig. 4:**
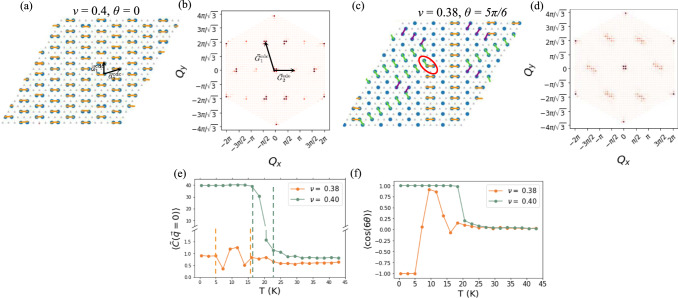
Columnar dimer crystal and type-II nematic states. **a** The columnar dimer crystal state obtained from Monte Carlo simulations at *ν* = 0.4, shown with nematic director orientation *θ* = 0. We annotate the nearest neighbor bonds and color them according to orientation. The red arrows indicate the columnar dimer crystal lattice vectors. **b** The Monte Carlo average of the structure factor at *ν* = 2/5, averaged over configurations with director orientation *θ* = 0. The red arrows indicate peaks at the reciprocal lattice vectors of the columnar dimer crystal state. **c** The fragmented dimer column state at *ν* = 0.38, shown with nematic director orientation *θ* = *π*/6. **d** The Monte Carlo average of the structure factor at *ν* = 0.38, averaged over configurations with director orientation *θ* = *π*/6. Broad peaks at the reciprocal lattice vectors of the generalized Wigner crystal state appear due to the short-range correlated regions of generalized Wigner crystal between the dimer column fragments. **e** The nematic order parameter correlation function is finite and constant at low temperatures, showing that these are nematic states. Error bars are smaller than the symbol size. We assign the critical temperature as midway between the dashed lines, and the dashed lines correspond to the error bars in Fig. 1d. **f** $\langle \cos (6\theta )\rangle$ for *ν* = 2/5 and *ν* = 0.38. The columnar dimer crystal has type-I nematicity as $\langle \cos (6\theta )\rangle=+ 1$ at low-T. The fragmented dimer column state is a type-II nematic as $\langle \cos (6\theta )\rangle=-\!1$ at low-T. Error bars are smaller than the symbol size.

Upon further diluting, the system maintains the same type of anisotropy and forms the columnar dimer crystal state at *ν* = 2/5, shown in Fig. 4a with director orientation *θ* = 0. This is the limit of the shortest stripe length, evolving from *ν* = 1/2. The *ν* = 2/5 state is a crystalline state with lattice vectors ${{{{{{{{\bf{a}}}}}}}}}_{1}^{{{{{{{{\rm{cdc}}}}}}}}}=(0,\sqrt{3})$ and ${{{{{{{{\bf{a}}}}}}}}}_{2}^{{{{{{{{\rm{cdc}}}}}}}}}=(5/2,\sqrt{3}/2)$. We mark the reciprocal lattice vectors ${{{{{{{{\bf{G}}}}}}}}}_{1}^{{{{{{{{\rm{cdc}}}}}}}}}=(-2\pi /5,\,\,2\pi /\sqrt{3})$ and ${{{{{{{{\bf{G}}}}}}}}}_{2}^{{{{{{{{\rm{cdc}}}}}}}}}=(4\pi /5,0)$ in the structure factor shown in Fig. 4b. Note that the peak at $2{{{{{{{{\bf{G}}}}}}}}}_{2}^{{{{{{{{\rm{cdc}}}}}}}}}$ is more intense than the one at ${{{{{{{{\bf{G}}}}}}}}}_{2}^{{{{{{{{\rm{cdc}}}}}}}}}$. This is due to the form factor from the lattice basis. As we dilute further, the length of the columns get shorter as dimers get broken up. At lower densities, the columns do not extend over the entire system, so there are finite length segments of columns that can have different orientations as illustrated in Fig. 4c. The broken pieces of dimers form short-range correlated domains of generalized WC. This is shown by the broad peaks in the structure factor in Fig. 4d. This compressible state no longer has the mirror symmetries of the columnar state. It is still anisotropic as we can see from the nematic correlation function in Fig. 4e. Interestingly, the columnar fragments intersect at 2*π*/3 angles, as well as *π*/3 angles, one of which is circled in red in Fig. 4c. While the 2*π*/3 intersections are isotropic, the *π*/3 intersections consist primarily of only two of the three possible nearest-neighbor bond orientations, and hence this state is a type-II nematic phase. We confirm this by observing that $\langle \cos (6\theta )\rangle=-1$ at low temperatures in Fig. 4f. (It is worth noting that although $\langle \cos (6\theta )\rangle$ gets very close to 1.0, it never actually reaches 1.0. We understand this to signify the growth of strong type-I nematic correlations near the phase transition to the type-II state. This could perhaps be due to the proximity to the columnar dimer crystal state.) Thus we predict the microscopic mechanism for the type-II nematic phase. As with the charge stripe and type-I nematic states, these transitions are first-order. In Fig. 1d, we again determine *T*_*c*_ and the error bars by jump center and width, respectively.

The nematic-II state in the region of 3/8 < *ν* < 2/5 is supported energetically. Upon increasing density beyond *ν* > 3/8, columnar fragments have to either intersect also at *π*/3 or be parallel to each other. Since the distance between columnar fragments increases away from the *π*/3 intersection, we expect *π*/3 intersections to be favored. See SI section 3 for a schematic calculation demonstrating this. Such *π*/3 intersections involve two nearest-neighbor bond orientations, promoting a nematic-II state.

## Discussion

One could experimentally probe our predicted nematic states by performing optical measurements similar to those done at *ν* = 1/2 in ref. 16. As one lowers the density from *ν* = 1/2 to *ν* = 1/3 we would anticipate a rotation of the nematic director and consequently a shift in the peaks of the measured optical anisotropy axis. In particular, as one decreases the density from between *ν* = 1/2 and *ν* = 2/5, we predict that the measured anisotropy axis should have peaks along the nematic-I orientations 0, *π*/3, 2*π*/3. Below *ν* = 2/5, when the director rotates into the nematic-II state, the peaks should be at *π*/6, *π*/2, 5*π*/6. Finally, below *ν* = 3/8 when the system becomes isotropic, we expect that there should be no preferred anisotropy axis at all. For 1/3 < *ν* < 3/8, one could also look for signatures of the hexagonal WC domain state using Umklapp spectroscopy experiments like those done in ref. 28. The short-range correlated nature of this state should show up as broadened Umklapp resonances around the *ν* = 1/3 generalized Wigner crystal lattice vectors. Recently developed techniques using scanning tunneling microscopy (STM)^12^ also provide a promising avenue for confirming our proposed phase diagram. The authors in ref. 12 have already had success in imaging the charge-ordered states at *ν* = 1/3 and 1/2. STM measurements in the intermediate density regime could ideally allow direct imaging of the compressible phases and produce results similar to our Monte Carlo configuration snapshots.

In summary, we studied the electronic states of a system of strongly correlated electrons on a triangular lattice in the region 1/3 ≤ *ν* ≤ 1/2 particles per Moiré site. At *ν* = 1/2, we find the charge stripe state. Upon dilution, the charge stripe state melts into a nematic-I short-ranged charge stripe state via the introduction of dislocations. Once the stripes become short enough, the columnar dimer crystal state emerges at *ν* = 2/5. At even lower densities, the remaining columnar fragments space themselves out to lower their energy by intersecting at *π*/3 and 2*π*/3 angles, resulting in a nematic-II state. Below *ν* = 3/8, the system can again lower its energy by using only 2*π*/3 columnar fragment intersections to form an isotropic, hexagonal network of domain walls between regions of the *ν* = 1/3 generalized Wigner crystal. Finally, at *ν* = 1/3, the pure, isotropic generalized Wigner crystal state emerges. We note that while the generalized Wigner crystal states are interaction-driven insulators, the compressibility experiments in ref. 16 suggest that this is not the case for the intermediate states. The system is found to be compressible at the densities at which the intermediate states occur. Our intermediate states were not only promoted by entropy, but we also found them to have lower energy compared to macroscopically phase separated states. Accordingly, we suspect that our proposed states are relevant for finite experimental temperatures where fluctuations due to entropy also play a role. We leave the determination of the classical ground state at *T* = 0 as a subject of future work.

As another subject of future work, we would like to consider the effects of a finite bandwidth in the TMD Moiré system. Although it is difficult to make a quantitative statement about how our proposed states would fare in the presence of a finite bandwidth from our classical simulations, we can draw some insight as to the relative stability of the phases from one energy scale available to us: *k*_*B*_*T*_*c*_. Estimating the hopping as *t* ~ 1 meV as in ref. 7 and comparing to the phase diagram in Fig. 1d, we anticipate the charge-ordered, type-I nematic, and hexagonal domain states to survive quantum fluctuations, but to observe the type-II nematic state might need further suppression of bandwidth. A quantum mechanical analysis using a technique such as DMRG is still needed to provide further detail, however.

Further support for our proposed phase diagram could be garnered by studying larger system sizes. Although a full finite-size scaling analysis is currently beyond our reach due to the runtime of the simulations for larger lattices, we are encouraged by the similarity of the results for smaller systems such as the *ℓ* = 10 system we consider in SI section 4.

Studying the intermediate phases of melted density waves has been of interest since considerations of Krypton adsorbed on graphene^19^. However, limitations in computational resources and experimental methods caused difficulties in probing the intermediate states. With advances in computing power and the advent of the TMD Moiré platform, however, detailed phase diagrams can now be predicted computationally and probed experimentally. Our work demonstrates this capacity to explore intermediate phases and the richness of the phase diagram one can obtain with a classical model, even without considering quantum effects. We found the striped phase predicted upon increasing density in ref. 19 refines into two distinct stripe crystal states neighboring two distinct types of nematics. In particular, we presented a microscopic mechanism for the formation of the nematic-II state via *π*/3 intersections between columnar fragments. As a subject of future work, it would be interesting to study the implications of our findings for the melting of WCs without a lattice potential, such as those recently observed in refs. 29, 30.

## Methods

Because the interaction Eq. (2) is long-ranged, simply simulating a system with periodic boundary conditions would result in ambiguous distance calculations. Thus, we simulate a formally infinite system that is constrained to be periodic in an *ℓ* × *ℓ* rhombus. Particles interact both within and between copies of the system. The choice of an *ℓ* × *ℓ* rhombus has the full symmetry of the triangular lattice as, when one considers the infinite system, the action of each element of the point group is a bijective map on the set of unique sites contained within the simulation cell. Thus we do not expect our choice of geometry to artificially promote rotational symmetry breaking. Moreover, in each nematic state that we report, our simulations find configurations with each of the three possible director orientations for the relevant nematic type with equal probability.

For Monte Carlo updates, we use arbitrary-range, single-particle occupancy exchanges with standard Metropolis acceptance rules. However, the prevalence of short-range correlated structures leading to long autocorrelation times complicates our simulations, especially in the incompressible density region. To get our simulations to converge, we need to perform cluster updates as well. Typical cluster update methods based on the Swendsen-Wang^31^ and Wolff^32^ algorithms for spin systems are insufficient for our needs as they do not simulate the correct ensemble. The geometric cluster algorithm^33^ does allow us to simulate a fixed number of occupied sites (i.e., the fixed magnetization ensemble of a spin model), but until now, has not been generalized to accommodate long-range interactions. Thus we develop our own cluster update method based on the geometric cluster algorithm that can handle arbitrary interactions. It is worth noting that our algorithm also works on an arbitrary lattice. For further details, see SI section 1. We perform a cluster update after every 1000 single particle occupancy exchange updates.

## Supplementary information

Supplementary Information

## Data Availability

The raw Monte Carlo data generated and analyzed for the current study have been deposited in a Zenodo repository, available publicly at ref. 34.

## Code availability

The code used to produce and analyze our results is available for download and use in our Github respoitory ref. 35.
