# Author Correction: Melting of generalized Wigner crystals in transition metal dichalcogenide heterobilayer Moiré systems

**DOI:** 10.1038/s41467-023-36457-5

**Published:** 2023-02-07

**Authors:** Michael Matty, Eun-Ah Kim

**Affiliations:** Department of Physics, Cornell University, Ithaca, NY 14853 USA

**Keywords:** Electronic properties and materials, Two-dimensional materials

Correction to: *Nature Communications* 10.1038/s41467-022-34683-x, published online 19 November 2022

The original version of this Article contained an error in the Acknowledgements, which incorrectly read ‘The authors acknowledge support by the NSF [Platform for the Accelerated Realization, Analysis, and Discovery of Interface Materials (PARADIM)] under cooperative agreement no. DMR-U638986.’. The correct version states ‘DMR-1539918’ in place of ‘DMR-U638986’. This has been corrected in both the PDF and HTML versions of the Article.

