# Supplementary material for: Melting of generalized Wigner crystals in transition metal dichalcogenide heterobilayer Moiré systems
Source: Nat Commun. 2022 Nov 19;13:7098. doi: 10.1038/s41467-022-34683-x (PMC9675862; doi:10.1038/s41467-022-34683-x)
Supplement: Supplementary file 1 — Supplementary Information [file 41467_2022_34683_MOESM1_ESM.pdf]

Melting of generalized Wigner crystals in transition metal dichalcogenide heterobilayer Moiré systems

Michael Matty, Eun-Ah Kim*
Department of Physics, Cornell University, Ithaca, New York 14853, USA
(Dated: October 10, 2022)

Supplementary Information

SECTION 1: CLUSTER ALGORITHM FOR MONTE CARLO SIMULATION

This appendix details the cluster algorithm we developed for our Monte Carlo simulations of the triangular Ising lattice gas with long range interactions. Our code is available in our group's Github repository: https://github.com/KimGroup/tmd_moire_monte_carlo. This algorithm is similar in spirit to the well-known Wolff algorithm [1] and its later generalization: the so-called geometric cluster algorithm used to simulate the fixed magnetization ensemble of the nearest-neighbor Ising model [2]. It is useful to consider the triangular lattice with N sites as having its sites indexed by integer values $i \in \mathbb{Z}_N$. We define an injective map $\mathcal{L} : \mathbb{Z}_N \to \mathbb{R}^2$ that takes an integer lattice site index and maps it to a real space coordinate. The precise action of this map depends on the details of the finite-size geometry. However, it will always return a linear combination of the triangular lattice vectors, i.e. $\mathcal{L}(i) = f(i)\vec{a}_1 + g(i)\vec{a}_2$ with lattice unit vectors $\vec{a}_{1,2}$ such that $\vec{a}_1 \cdot \vec{a}_2 = \pm a^2/2$ for some integer valued functions f, g and where $a \in \mathbb{R}^+$ is the lattice constant.

One can view a particle configuration as a set $\Lambda \subseteq \mathbb{Z}_N$ specifying occupied sites of the lattice. The Lattice is occupied by Ising variables, and the occupancy function $n : \mathbb{Z}_N \times \mathbb{Z}_N \to \mathbb{Z}_2$ acts on a site index i and configuration Λ as

$$n(\Lambda, i) = \begin{cases} 1 & i \in \Lambda \\ 0 & \text{otherwise} \end{cases}.$$

We are interested in the case of a fixed number of particles $M \leq N$, i.e. the only valid particle configurations Λ are those with cardinality M.

We treat elements of the point group $\tau \in \mathcal{G}$ as maps $\tau : \mathbb{Z}_N \to \mathbb{Z}_N$ which map lattice site indices to lattice site indices. Particle exchange is a map $\eta : \mathbb{Z}_N \times \mathbb{Z}_N \times \mathcal{G} \to \mathbb{Z}_N$ defined as

$$\eta(\Lambda, i, \tau) = \begin{cases} \Lambda & n(\Lambda, i) = n(\Lambda, \tau(i)) \\ (\Lambda \setminus \{i\}) \cup \{\tau(i)\} & i \in \Lambda, \tau(i) \notin \Lambda \\ (\Lambda \setminus \{\tau(i)\}) \cup \{i\} & \text{otherwise} \end{cases}.$$

Note that η preserves the cardinality of Λ.

The Hamiltonian for the system is $\mathcal{H}(\Lambda) = \frac{1}{2} \sum_{i \neq j} V_{ij} n(\Lambda, i) n(\Lambda, j)$ where $V_{ij} = V(|\mathcal{L}(i) - \mathcal{L}(j)|)$.

Algorithm:

1. Fix a particle configuration Λ, and initialize an empty set $\mathcal{C}$ (the cluster)
2. Choose an order-2 element, τ^*, of the lattice point group.
3. Randomly choose a site i, set $\mathcal{C} = \mathcal{C} \cup \{i, \tau^*(i)\}$ and $\Lambda' = \eta(\Lambda, i, \tau^*)$
4. For each other site k with $V_{ik} \neq 0$ and $k \notin \mathcal{C}$:

   (a) Calculate

   $$\Delta_{ik}(\Lambda) \equiv \frac{1}{2}[n(\Lambda, i) - n(\Lambda, \tau^*(i))][n(\Lambda, k) - n(\Lambda, \tau^*(k))][V_{\tau^*(i),k} - V_{i,k}].$$

   The form of Δ_{ik} is chosen to ensure detailed balance (more detail in the next section).

(b) With probability $\max\left(0, 1 - e^{-\beta\Delta_{ik}(\Lambda)}\right)$, set $\mathcal{C} = \mathcal{C} \cup \{k, \tau^*(k)\}$, $\Lambda' = \eta(\Lambda', k, \tau^*)$, and record k in a stack data structure

5. Pop an element j from the stack and repeat step 4 with j playing the role of i

6. Repeat step 5 until the stack is empty and then return the updated particle configuration Λ'.

Proof of Detailed Balance:

To prove that this generates the correct equilibrium probability distribution (Boltzmann distribution), we need to show that the probability of a particle configuration Λ moving to a configuration Λ', $\mathcal{P}(\Lambda \to \Lambda')$, satisfies the detailed balance condition, i.e.

$$\frac{\mathcal{P}(\Lambda \to \Lambda')}{\mathcal{P}(\Lambda' \to \Lambda)} = e^{-\beta(\mathcal{H}(\Lambda') - \mathcal{H}(\Lambda))}. \tag{1}$$

Observe that, for a move corresponding to a cluster $\mathcal{C}$, we can write $\mathcal{P}(\Lambda \to \Lambda') = \mathcal{P}_{\text{in}}(\mathcal{C}, \Lambda)\mathcal{P}_{\text{out}}(\mathcal{C}, \Lambda)$ where the first factor is the probability of forming the cluster containing the sites in $\mathcal{C}$ and the second is the probability that no sites in $\mathbb{Z}_N \setminus \mathcal{C}$ are included in $\mathcal{C}$. Similarly, we write $\mathcal{P}(\Lambda' \to \Lambda) = \mathcal{P}_{\text{in}}(\mathcal{C}, \Lambda')\mathcal{P}_{\text{out}}(\mathcal{C}, \Lambda')$.

Because (1) τ^* is order-2 and a symmetry of $\mathcal{H}$ and (2) $\mathcal{P}_{\text{in}}(\mathcal{C}, \Lambda)$ only depends on lattice sites in $\mathcal{C}$, we have that $\mathcal{P}_{\text{in}}(\mathcal{C}, \Lambda) = \mathcal{P}_{\text{in}}(\mathcal{C}, \Lambda')$.

By construction of the algorithm, we have (using standard probability rules) that

$$\mathcal{P}_{\text{out}}(\mathcal{C}, \Lambda) = \exp\left(-\beta\frac{1}{2}\sum_{i\in\mathcal{C}}\sum_{k\notin\mathcal{C}}\max(0, \Delta_{ik}(\Lambda))\right)$$

and

$$\mathcal{P}_{\text{out}}(\mathcal{C}, \Lambda') = \exp\left(-\beta\frac{1}{2}\sum_{i\in\mathcal{C}}\sum_{k\notin\mathcal{C}}\max(0, -\Delta_{ik}(\Lambda))\right).$$

where in the last equation we have used the fact that for $i \in \mathcal{C}$, $n(\Lambda', i) = n(\Lambda, \tau^*(i))$. Thus we have that

$$\frac{\mathcal{P}_{\text{out}}(\mathcal{C}, \Lambda)}{\mathcal{P}_{\text{out}}(\mathcal{C}, \Lambda')} = \exp\left(-\beta\frac{1}{2}\sum_{i\in\mathcal{C}}\sum_{k\notin\mathcal{C}}\Delta_{ik}(\Lambda)\right) = \exp\left(-\beta\frac{1}{2}\sum_{i\in\mathcal{C}}\sum_{k\notin\mathcal{C}}n(\Lambda, i)n(\Lambda, k)[V_{k,\tau^*(i)} - V_{k,i}]\right) \tag{2}$$

where in the second equality we have used that τ^* is order-2 and a symmetry of the Hamiltonian. Note also the factor of $1/2$, which is due to the fact that since $i \in \mathcal{C} \Leftrightarrow \tau^*(i) \in \mathcal{C}$ and $\Delta_{ik}(\Lambda) = \Delta_{\tau^*(i)k}(\Lambda)$ the sum double counts.

What remains is to show that the RHS of eq. 1 is the same as eq. 2. Using the fact that τ^* is order-2 and a symmetry of the Hamiltonian, we can write

$$\mathcal{H}(\Lambda') = \frac{1}{2}\sum_{x\neq y, x,y\notin\mathcal{C}} n(\Lambda, x)n(\Lambda, y)V_{x,y} + \frac{1}{2}\sum_{i\neq j, i,j\in\mathcal{C}} n(\Lambda, i)n(\Lambda, j)V_{i,j} + \sum_{i\in\mathcal{C}}\sum_{x\notin\mathcal{C}} n(\Lambda, x)n(\Lambda, i)V_{x,\tau(i)}.$$

It is then easy to see that the difference

$$\mathcal{H}(\Lambda') - \mathcal{H}(\Lambda) = \sum_{i\in\mathcal{C}}\sum_{x\notin\mathcal{C}} n(\Lambda, x)n(\Lambda, i)[V_{x,\tau^*(i)} - V_{x,i}].$$

This completes the proof and establishes that the algorithm defined above satisfies detailed balance.

A Note about Ergodicity:
Note that by choosing τ^* as an appropriate reflection, it is possible to form a cluster consisting of a nearest neighbor particle exchange with finite probability. Therefore, it is possible with finite probability to get from any particle configuration to any other in the same way that it is possible to realize any spin configuration in an Ising model via a sequence of single spin flips. Thus we can see that this algorithm is indeed ergodic.

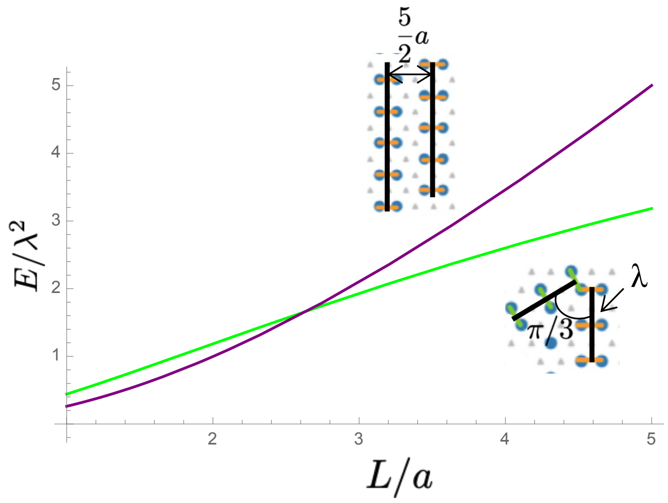

Supplementary Fig. 1. **Wire Energies.** Energy of isolated, continuous wires interacting via eq. **??** with uniform line charge density λ as a function of wire length L. We show results for parallel wires seperated by $5a/2$ (purple) and wires angled at $\pi/3$ with minimum separation $\sqrt{3}a/2$ (green). For sufficiently long wires the angled wires have lower energy.

SECTION 2: CALCULATION OF NEMATIC CORRELATION FUNCTION AND ORIENTATION

To calculate the nematic order parameter expectation value $\langle N(\vec{r})\rangle = \langle n(\vec{r})e^{i2\theta(\vec{r})}\rangle$ in Monte Carlo, we write $N(\vec{r})$ as

$$\vec{N}(\vec{r}) = \sum_{\vec{\delta}} \rho(\vec{r})\rho(\vec{r}+\vec{\delta}) \begin{pmatrix} \delta_x^2 - \delta_y^2 \\ 2\delta_x\delta_y \end{pmatrix} \tag{3}$$

where the vectors $\vec{\delta}$ point to the nearest neighbors of $\vec{r}$ and $\rho(\vec{r})$ is the occupation number of the site at $\vec{r}$. This is easy to calculate directly from a Monte Carlo configuration and average over the Markov chain. To calculate the orientation $\langle\cos(6\theta)\rangle$ we use the fact that we can write the vector part of the nematic order parameter as $(\cos(2\theta), \sin(2\theta))^T$. From this we can simply calculate θ and hence $\cos(6\theta)$ from the nematic order parameter and average it over the Markov chain.

SECTION 3: ENERGETICS OF $\pi/3$ INTERSECTION AND PARALLEL DIMER COLUMN FRAGMENTS

We can understand why the $\pi/3$ intersections are energetically preferred schematically by calculating the energy for two continuous, isolated wires of uniform line charge density λ interacting via eq. **??** as a function of wire length L. We approximate the wires as being at the center of the dimer columns, and plot the results for parallel wires separated by $5a/2$ in the purrple curve in Fig. S1. For a $\pi/3$ intersection with wires at the center of the dimers, the wires terminate with a separation of $\sqrt{3}a/2$. We show the results for such wires in the green curve in Fig. S1. Indeed, the energy of the parallel wires exceeds that of the angled wires for sufficient L.

SECTION 4: THE SYSTEM SIZE DEPENDENCES

Here we compare simulations on the $\ell = 20$ lattice to simulations on a smaller, $\ell = 10$ lattice. At $\ell = 10$, the $2\pi/3$ domain wall junction appears at $\nu = 0.36$ in Fig. 2(a). This is the defining feature of the hexagonal domain wall state. However, the system is still not large enough to fit an entire hexagonal domain. Increasing the density to $\nu = 0.38$ in Fig. 2(b), we can see that $\pi/3$ intersections begin to emerge as we would expect for the nematic-II state, but the system is still too small to fit longer columnar fragments. Finally, in Fig 2(c) at $\nu = 0.48$, the simulation cell isn't big enough to fit stripe fragments and dislocations in a single orientation, and instead forms two domains of stripe fragments. What is encouraging about the results from the $\ell = 10$ system is that the defining features of the

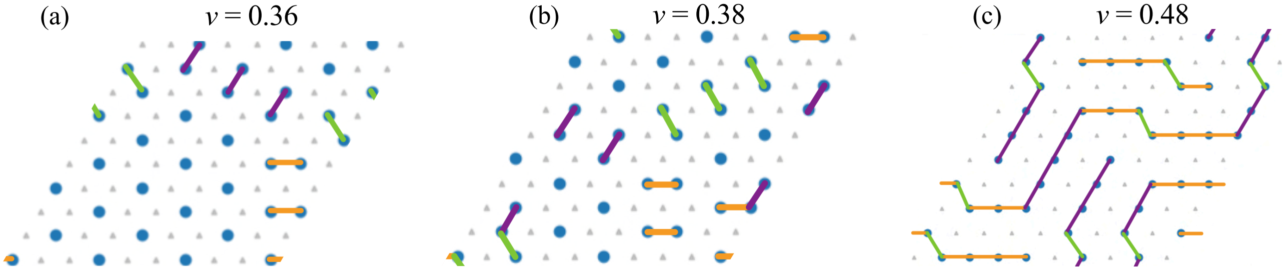

Supplementary Fig. 2. **Results from $\ell = 10$ system.** Low-temperature Monte Carlo snapshots from an $\ell = 10$ system at densities corresponding to the intermediate phases observed in the larger $\ell = 20$ system (c.f. main text Fig. 1(d)). (a) $\nu = 0.36$ corresponding to the hexagonal domain wall state (b) $\nu = 0.38$ corresponding to the type-II nematic state (c) $\nu = 0.48$ corresponding to the type-I nematic state.

intermediate states show up as long as they fit in the smaller system. However, if the system size is too small for a particular structure to show up (e.g. dislocations), it may not be visible.

* eun-ah.kim@cornell.edu

[1] Ulli Wolff, "Collective Monte Carlo Updating for Spin Systems," Phys. Rev. Lett. **62**, 361–364 (1989).
[2] J. R. Heringa and H. W. J. Blöte, "Geometric cluster Monte Carlo simulation," Phys. Rev. E **57**, 4976–4978 (1998).
